# Priority Setting for the Management of Chemicals Using the Globally Harmonized System and Multivariate Analysis: Use of the Mahalanobis-Taguchi System

**DOI:** 10.3390/ijerph16173119

**Published:** 2019-08-27

**Authors:** Hong Lyuer Lim, Eun-Hae Huh, Da-An Huh, Jong-Ryeul Sohn, Kyong Whan Moon

**Affiliations:** 1 Department of Health and Safety Convergence Science, Korea University, Anam-ro 145, Seongbuk-gu, Seoul 02841, Korea; 2 Department of Health Science, Korea University, Anam-ro 145, Seongbuk-gu, Seoul 02841, Korea

**Keywords:** chemical ranking and scoring, Globally Harmonized System, Mahalanobis–Taguchi System, priority setting

## Abstract

This study aims to provide a new methodology using the Globally Harmonized System (GHS) and the Mahalanobis–Taguchi System (MTS) that can be used to assess the overall hazard of a chemical using GHS information. Previously, hazardous chemicals were designated and managed by the Chemical Management Act, but many more chemicals are now in use. Damage prediction modeling programs predict the extent of damage and proactively manage high-risk chemicals, but the lack of physical and chemical characterization information relating to chemicals has limitations that cannot be modeled. To overcome such limitations, a new method of chemical management prioritization was developed using the GHS and Mahalanobis–Taguchi System (MTS). For effective management, the risk of a chemical can be ranked according to a comprehensive risk assessment and calculated through multivariate analysis using the GHS. Relative hazards are then identified using MTS multivariate analysis with GHS information, even when there is insufficient information about the chemical’s characteristics, and the method can be applied to a large number of different chemicals.

## 1. Introduction

With the increased expansion of industries, there has been a corresponding increase in the number of chemicals stored, handled, and distributed. More than 44,000 types of chemicals are circulating in Korea and more than 300 new chemicals are introduced each year [1,2]. The number of chemical accidents has increased in line with the increased usage of chemicals. For example, in 2012, a hydrogen fluoride leakage accident in Gumi, North Gyeongsang Province resulted in damages worth more than 50 billion, caused the death of five people, and injured 18, in addition to damaging crops and livestock. A series of chemical accidents has thus raised the public’s interest about the safe management and use of chemicals [3,4].

In Korea, chemicals that are considered highly likely to cause chemical accidents due to their hazardous nature, or which are likely to cause severe damage if an associated accident occurs, are designated as “chemicals requiring preparation for accident” and require an accident preparation plan to be devised under the Chemical Control Act, with the aim of preventing such damage from chemical accidents [5,6,7]. In this respect, the damage prediction modeling program provides preferential management of high-risk chemicals by predicting the extent of damage that would be caused by chemical accidents [8,9]. However, under the Chemical Control Act, fewer than 100 types of substances are controlled and designated as “chemicals requiring preparation for accident”, which is a very small number compared to the actual number of chemicals currently in use. In addition, the damage prediction modeling program has a limitation whereby it is not possible to employ modeling if information pertaining to the physical and chemical properties of the chemical is missing. For example, a modeling program developed by U.S. EPA, ALOHA, provides physical and chemical data of only 1,000 types of chemicals in its own chemical library [10].

To prevent chemical accidents, it is necessary that a greater number and variety of chemicals are assessed and managed. However, the numbers actually assessed are low, because it takes a considerable amount of time and resources to identify the overall hazard of a chemical [11]. In addition, it is difficult to obtain consistent information even about one particular chemical, because of the varying degrees of hazards expressed in different studies. The Globally Harmonized System (GHS) can provide uniform chemical information and information about chemical-physical properties and associated health hazards [12]. It is also expected that the GHS can be used to assess chemical hazards more proficiently than existing methodologies, as GHS information is relatively readily available.

Therefore, in this study, we propose the use of GHS information to develop a new methodology for assessing the overall hazard of a chemical. The multivariate analysis, Mahalanobis–Taguchi System (MTS) is employed, to simultaneously consider the various hazard variables provided by the GHS. This statistical technique can deliver the following: present multivariate data as a comprehensive indicator, provide the Mahalanobis distance (MD), and simultaneously consider the correlation between data. As variables affecting the hazard of a chemical vary and there is a correlation between these variables, it is appropriate to assess the overall hazard of a chemical using the MTS. We used existing studies using MTS methodologies, such as scoring method, MD, etc. to set up research methods. The new method is used in this study to prioritize chemicals and the results are compared with those of a modeling program previously employed in chemical management. A sensitivity analysis is also used to verify the reliability of the new methods. It is expected that this new methodology can be applied to more chemicals than in existing methods, with the aim of providing efficient and effective chemical management.

## 2. Materials and Methods

### 2.1. Mahalanobis–Taguchi System (MTS)

The MD was introduced by the Indian statistician, Mahalanobis, to measure the distance between a point and a distribution. Taguchi, who devised the associated structural design method, set the Mahalanobis Space (MS) based on the average value of a group, and measured the distance between new observations and the MS. The Mahalanobis–Taguchi System (MTS) defines the MS within a multi-dimensional space and uses MD to indicate the distance between a random target and the MS.

Numerical information data is required to calculate the MD. Factors are used to consider and identify the relative differences between the reference group and any measured group in the reference space, MS, are referred to as variables in MD calculations and are expressed as X_1_, X_2_, …, X_k_ (Table 1). For each substance of which the reference and measured group is comprised, information corresponding to the number of variables is quantified. As the range of numeric values varies depending on the variables employed, to enable equal comparisons to be made, the information for each variable is replaced by a standardized score with a mean of zero and a standard deviation of one.

Using the standardized scores obtained in this way, the $c_{ij}=\frac{1}{n}\overset{n}{\underset{l=1}{\sum }}z_{il}\cdot z_{jl}$ method calculates the correlation coefficient and obtains the correlation matrix.

$$z_{ij}=\frac{x_{ij}-\bar{x_{i}}}{s_{i}},\;\mathrm{where}\;\bar{x_{i}}=\frac{1}{n}\left(x_{i1}+x_{i2}+\cdots +x_{in}\right),\;\mathrm{and}\;s_{i}=\sqrt{\frac{\sum_{j=1}^{n}{\left(x_{ij}-\bar{x_{i}}\right)}^{2}}{n-1}}.$$

After this process, the MD of the j_th_ material can be calculated as $MD_{j}=\frac{1}{k}\cdot Z_{j}^{T}\cdot C^{-1}\cdot Z_{j}$, where $Z_{j}=\left(z_{1j},\;z_{2j},\cdots ,z_{kj}\right)$ means the standardized variable vector of the j_th_ matter [13,14,15,16,17,18,19,20].

The MTS can be extended by combining the MD with Taguchi’s orthogonal array; this array is used to reduce the number of variables in multivariate systems. This study was only concerned with the process of calculating the ranking of chemical health hazards using the MD and its reliability verification.

### 2.2. Calculation of Mahalanobis Distance (MD) using the Globally Harmonized System (GHS)

#### 2.2.1. Globally Harmonized System (GHS)

The GHS provides information on 16 chemical-physical property variable and 15 health hazard variables. Each variable provides classified information, “Category”. “Category” divides different levels depending on each variable. The more hazardous the classification information, the nearer it is to “Category 1”. If a chemical has no GHS information, it is considered as “Classification Not Possible” in the GHS and “Not Classified” if the chemical status does not conform to a hazardous variable defined in the GHS [12].

This study used GHS classification information for 3028 out of the 3967 chemicals provided by the National Institute of Technology and Evaluation (NITE), excluding those that did not have or had an overlapping Chemical Abstract Service (CAS) registry number. Among the physical and chemical characteristics variables, hazard information was provided for Flammable, Oxidizing, and Pyrophoric variables depending on whether the state of the material was solid, liquid, or gas. Among the health hazards variables, the Acute toxicity (Inhalation) variable was subdivided into gas, vapor, dust, and mist. In this study, the variables Flammable solid, Flammable liquid, and Flammable gas were incorporated into the most hazardous values, making them one variable, as were the variables Oxidizing and Pyrophoric. The variables Acute toxicity(inhalation) gas, vapor, dust, and mist were incorporated into the most hazardous values, making them one variable. In addition, three variables were excluded(respiratory sensitivities, skin sensitivities, and inhalation hazards) because their ratios of “Classification Not Possible” or “Not Applicable” were greater than 70%. Finally, seven physical and ten health hazards were selected, as shown in Table 2, and a total of 17 variables were employed to calculate the overall hazard of the chemical.

#### 2.2.2. Construction of the Mahalanobis Space (MS)

The first step in calculating the MD is to set up a standard space called the MS using objects belonging to the reference group, which is composed of relatively safe chemicals. Since all chemicals have a certain level of hazard, a standard space should be constructed using substances that are considered to be relatively less harmful.

In this study, the following criteria were applied to select chemicals that are relatively less hazardous: First, we excluded substances that were classified more than once as being in the most harmful category, “Category 1”. Second, we excluded substances that had more than two “Category 2” classifications. Thirdly, any chemical that had a “Category 2” health hazard variable including carcinogenic, reproductive toxicity, and reproductive cell mutagenic variables was excluded. Fourth, substances that had more than eight “Classification Not Possible” values were excluded. According to this standard, a total of 114 chemicals of the 3028 chemicals were selected for incorporation into the reference groups, and the substances were then used to build a Mahalanobis space. Table 3 shows the process of selecting chemicals for reference groups that are relatively less harmful.

#### 2.2.3. Scoring of Hazard Variables

The GHS classification was quantified to calculate the MD of the 3028 chemicals. In this study, each variable was graded according to the Globally Harmonized System of Classification and Labelling of Chemicals (GHS). The GHS details the classification criteria for each variable. Regardless of variables, the most harmful, Category 1, was given a score of 50 and smaller scores indicated a lower hazard. “Not Applicable” was assigned the lowest score of 1 and “Not classified” was assigned 25 points, which is lower than the score of Category 5–7. For “Classification Not Possible,” 42 points were allocated between Category 2 and 3, because an absence of data does not mean that the chemical is safe. Table 4 shows the results of health hazard quantification.

### 2.3. Sensitivity Analysis of MD Rank

Even if the same variables are used in calculation, MD values can vary depending on how these variables are quantified. Therefore, it is necessary to test that the MD calculation method is reliable. To obtain and compare results in this study, three scenarios were established that gave different weights to variables. In scenario A, the method of scoring classification information was adjusted, and we define the scoring method in Table 4 as scenario A2. A1 narrowed the gap between scores and A3 made the gap wider. Secondly, in Scenario B, the score of “Classification Not Possible” was adjusted. B1 set the score for “Classification Not Possible” to 15 points, which is the score between “Category 1” and “Category 2”; A2 set the score for “Classification Not Possible” to 42 points, which is the score between “Category 2” and “Category 3”; B2 set the score for “Classification Not Possible” to 37 points, which is the score between “Category 3” and “Category 4”; B3 set the score for “Classification Not Possible” to 32 points, which is the score between “Category 4” and “Category 5–7”; and B4 set the score for “Classification Not Possible” to 27 points, which is the score between “Category 5–7” and “Not Classified”. Third, Scenario C adjusted the score of “Not Applicable” based on A2, which assigned 1 point to “Not Applicable”. In each scoring method in scenario C, we assigned the score for “Not Applicable” as follows: A total of 3 points were allocated for C1, 5 points for C2, 7 points for C3, and 9 points for C4. Table 5 shows the quantification results of hazard variables using the three scenarios.

### 2.4. Comparison with Results of Damage Prediction Modeling Program

#### 2.4.1. ALOHA (Areal Locations of Hazardous Atmospheres)

To test the reliability of the chemical ranking results using the MD, the results and matching rates were compared using the damage prediction modeling program. The ALOHA program provided by the US EPA was used as a comparison. ALOHA is used for rapid emergency planning in the event of chemical leakage incidents. ALOHA has its own chemical library with the physical characteristics of approximately 1,000 chemicals that have common risks; therefore, users do not need to enter such data.

ALOHA enters data details for actual or potential chemicals and calculates the Threat Zone for different types of hazards and ALOHA can be used to model various situational types, such as a Toxic Gas Cloud, Flammable Gas Cloud, BLEEVEs (Boiling Liquid Expanding Vapor Deposition), Jet Fire, and a Fire Pool [21,22].

Although the ALOHA program is somewhat less accurate than programs that require payment to use, it is commonly employed in the industry because it is free to use, its modeling results are easily expressed on maps, and it provides continuous automatic updates.

#### 2.4.2. Establishing a Virtual Accident Scenario to Determine Estimated Damage Distance

In this study, a virtual accident scenario was established to determine the expected damage distance in the event of a chemical accident. The accident site selected was a plant located in the industrial complex area in Incheon, Korea.

Weather variable conditions for the average air temperature and average wind speed were based on the National Climate Data Service Center (NCDSC). Ground weather observation data was obtained from the Korea Meteorological Administration.

The storage tank was assumed to be a vertical circular cylinder with a diameter of 3.1 m and a height of 4 m and the chemical occupied 80% of the tank. Leakage occurred at a height equal to 10% of the total height from the floor and the diameter of the leakage ball was set as 5 cm.

**Figure 1:**
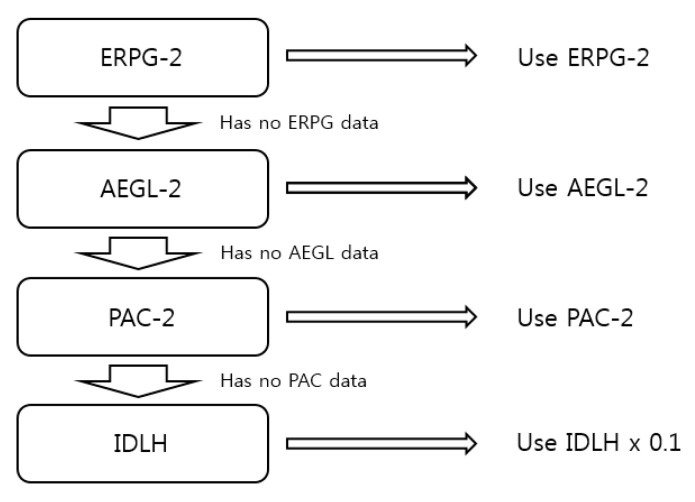
Endpoint concentration criteria.

In this study, the endpoint concentration was calculated using the Emergency Response Planning Guide (ERPG-2) value when the ERPG-2 value was available. If ERPG-2 was not available, the AEGL-2, PAC-2, and IDLH values were converted to criteria as in Figure 1.

#### 2.4.3. Selection of Chemicals to be Compared

To compare the results of the MTS between the programs, 60 substances out of the 97 chemicals that were “chemicals requiring preparation for accident” (as designated by the Chemical Control Act) were selected for modeling [7].

### 2.5. Statistical Analysis

The correlations between variables used in the MD calculation were analyzed and a Spearman correlation analysis was conducted to determine whether the chemical ranking was consistent. The Kappa statistic was also employed to compare the levels of grouped chemicals. The Statistic Package Program Minitab 18 developed by Minitab Inc. was used to produce the MD and IBM SPSS Statistics v25.0 was used to calculate the other statistics.

## 3. Results

### 3.1. Hazard Priority Setting Using MTS

**Figure 2:**
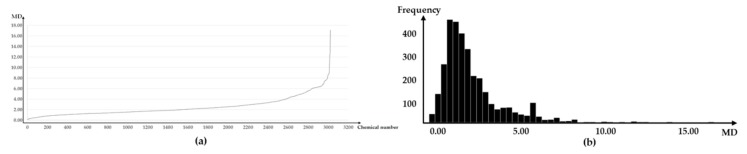
MD distribution of 3028 chemicals: (**a**) Distribution of MDs in ascending order; and (**b**) frequency distribution of MDs.

Figure 2 shows the MD distribution for the 3028 chemicals. The MD ranged from a minimum of 0.134 to a maximum of 17.093 and a geometric distribution was evident when values were placed in an ascending order. In distribution of MD ascending order, chemical’s MDs that have a MD under 6 gradually increased and chemical’s MDs that have a MD over 6 increased dramatically. With respect to the MD values of all chemicals that are skewed to the left, over 90% of the 3,028 chemicals had an MD value of 5 or less and 1% of the chemicals were found to have an MD value of 8 or more.

The largest MD values of the 3028 chemicals were attributed to butyl lithium (MD = 17.093) and pentaborane (MD = 14.644). Chemicals with high MD values were in high risk categories for the three properties of flammable, spontaneous ignition, and water reactivity (of the physical-chemical properties reflected in MD calculations). Of the top 10 chemicals, sufficient information was available from the physical chemistry characteristics to enable simulations using ALOHA and two substances were managed by the Chemical Control Act.

### 3.2. Sensitivity Analysis Results

Table 6 shows the degree of correlation between the MD ranking using each of the scenarios, where scenarios A and C show the highest correlations of 0.9, whereas Scenario B shows a drastic reduction in the correlation between B1–B4 and between both scenarios A and C.

### 3.3. Chemical Ranking Comparison Using MD and Damage Distance

**Figure 3:**
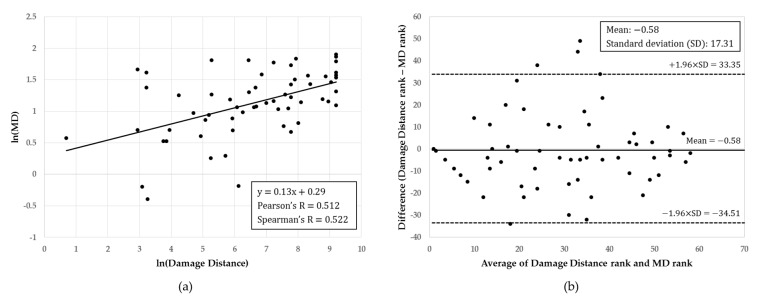
Comparison of matched ratio between MD and Damage Distance for 60 chemicals that require an accident plan: (**a**) Comparison between MD and Damage Distance and (**b**) Bland–Altman plot of MD and Damage Distance.

Figure 3 shows the results of an analysis conducted on the matched ratio between the MD and damage distance. The ranking of chemicals calculated using the MD and damage distances correlates to positive linearity and the Spearman correlation coefficient between the two ranks is r = 0.522. For the Bland–Altman plot, only four out of the 60 data are outside the 95% confidence interval.

Table 7 shows the results of analyzing the matching rate after grouping the chemical rankings. The highest correlation was found between the groupings when the rankings were grouped using the geometric interval classification and the quadratic weighted Kappa statistics range was between 0.380 and 0.741 (where Kappa statistics reflect an increasing tendency in accordance with a growth in weight).

## 4. Discussion

This study employs GHS information and MTS multivariate analysis to present and validate a new methodology that can be used to assess the overall hazard of chemicals. Chemicals that are highly flammable, are self-heating, and react with water tend to have larger MDs (out of all other physical and chemical properties). The method of scoring GHS information was altered to conduct a sensitivity analysis and the results showed that the MD ranking of chemicals was similar, except when a change was made to the scoring of “Not Classified”. The chemical ranking calculated using the MD and damage distance, respectively, showed a matched rate of 0.741 (quadratic weighted Kappa = 0.741).

Table 5 presents the use of three scoring scenarios. Scenario A represents MD ranking when varying the width of scores given by the classification based on the A2 quantification method. Scenario C only changes scores given to “Not applicable” based on the A2 quantification method in Scenario A. As the score increased from C1 to C4, the change in MD ranking was analyzed. When applying the three methods assumed in Scenario A, A1, A2, and A3, the Spearman correlation coefficient between each MD rank was 0.978, and that between the MD rank applied with C1, C2, C3, and C4 for scenario C was 0.996 or higher. These results show that there were no significant differences in the MD standings when quantification methods of Scenarios A and C were employed and that any change in the width of the scores by category or any other value applied to the “Not Applicable” score did not significantly affect the MD ranking. In Table 5, the ranking correlation between A1 to A3 in Scenario A and C4 in Scenario C was higher than 0.981. This result means that the ranking between different scenarios has a consistency greater than 98%. Therefore, different quantification methods employed by various researchers will provide similar results.

However, scenario B changed the “Classification not possible” score based on A2 and according to the results of Table 6, it is difficult to maintain a similar MD ranking when this score is changed. In scenario B, there is a greater reduction in the consistency of the ranking and matching rates as scenario B1 goes to B4. This result shows that the MD rankings differ significantly when differing “Classification not possible” scores are employed. The effect of the “Classification not possible” quantification method has been discussed in previous studies. According to Yoko Kubota’s study, giving a “Classification not possible” score that is similar to “Category 2” may overestimate the effect of “Classification not possible” and is most appropriate when giving a score close to “Category 3” [23,24]. In this study, therefore, the results were calculated by setting “Classification not possible” to 42 points and close to “Category 3”.

The top 10 chemicals with large MDs contained highly hazardous substances with respect to the three physical chemistry properties (being flammable, pyrophoric, and reacting to water) and these are reflected in the MD calculations. Nine out of the 10 substances were highly flammable or pyrophoric and four of the top 10 substances that were considered to be high risk were both flammable and pyrophoric. Four substances were highly reactive to water and produced flammable gases in response to water contact and they all had high pyrophoric properties. Butyl Lithium ranked No. 1 in the rankings and was labelled as follows: Flammable “Category 2”, pyrophoric “Category 1”, and water reactivity “Category 1”. As such, butyl lithium is considered to be a high risk with respect to all three major physical-chemical properties of top-ranked chemicals in the MD rankings. The second-ranked, pentaborane, was labelled fire-resistant “Category 3” and pyrophoric “Category 1”. 

Of the top 10 chemicals with large MDs, the controlled chemicals were carbon monoxide and phosphorus (yellow), both of which are designated by the Chemical Control Act. The other eight substances escaped legal control, even though they were classified by multivariate analysis as being the 10 most dangerous substances out of 3000. Even if the MD was expanded to include the top 100 chemicals, only 20 substances would be designated and managed by the Chemical Substance Control Act. This shows that a significant number of hazardous substances are not properly managed. In fact, there are no more than 1000 chemicals that are designated and managed under the Chemical Control Act in Korea, and these include “chemicals requiring preparation for accident”, “toxic chemicals”, “prohibited chemicals”, and “restricted chemicals” [7]. In addition, only five of the top 10 materials with large MDs are currently included in the ALOHA chemical database and can thus be simulated. As there are now more than 40,000 types of chemicals circulating in Korea, it is evident that many more substances need to be adequately managed [1,2].

This study is a follow-up paper to “Development of a Screening Method for Health Hazard Ranking and Scoring of Chemicals Using the Mahalanobis–Taguchi System”, and the comparative analysis with the prior study was done sufficiently in that paper. In addition, the main purpose of this study was to verify the completeness and reliability of the change in the ranking of the harmfulness under several conditions, after calculating the ranking of chemical hazards using MTS in “Development of a Screening Method for Health Hazard Ranking and Scoring of Chemicals Using the Mahalanobis–Taguchi System”. The literature and this study are commonly used to assess the harmful effects of chemicals by using GHS information and MTS multivariate analysis. 

The literature proposed a new methodology and tested its usability, compared with the existing screening and scoring methods. In this study, the new methods presented in the research were applied to “chemicals requiring preparation for accident,” designated and managed by the Korean Chemical Management Act. The harmonization rate was confirmed compared with the damage resistance result, which was previously used as a reference in determining the harmfulness of “chemicals requiring preparation for accident.” In this regard, this study should be viewed as a follow-up to the extension of the literature. There are three main differences between the literature and this study, as follows:

First, the physical and chemical properties of chemicals were not applied in the existing paper. Second, the current article compared the results with methodologies that only evaluated the health hazards of chemicals. However, there is a big difference in that this study compared results with damage distances that reflect both physical and health hazards. Third, the sensitivity analysis was performed and verified to assess the reliability of the chemical hazard priority results using MD.

Prioritization of 60 chemicals among “chemicals requiring preparation for accident,” calculated using MDs and damage distances, were found to have a positive linear correlation, as seen in Figure 3a, which was relatively high with Spearman’s value of r = 0.522. This can also be found in Figure 3b, which includes all data (except four data) that are within the 95% confidence interval range using the mean of the MD and the distance difference. The quadratic weighted Kappa statistics, which grouped the chemicals using the geometric interval classification, was 0.741. This reflects the high MD ranking of 60 chemicals of “chemicals requiring preparation for accident”. It is thus evident that reliable results can be obtained using multivariate analysis with the GHS to manage chemicals and it is believed that the use of complementary methods, such as damage prediction modeling programs, can be used in conjunction with the method proposed here to manage existing chemicals more effectively.

This study presents a new methodology for prioritizing chemical management using chemical GHS information and the MD and compares the results with an existing methodology. The reliability of the new method was proven by analyzing the multivariate values presented in this study and the internal validity was verified using various scenarios. Similar results were found for all scenarios, except for cases where the GHS scores were changed for “Classification not possible”.

## 5. Conclusions

This study presented a new methodology for establishing chemical management priorities using GHS and multivariate analysis. The MTS multivariate analysis method produced the MD by considering the various chemical-physical and health hazard properties of various chemicals. Employing the new methodology presented in this study may complement the limitations of chemical management relating to existing damage prediction modeling programs or systems such as the Chemical Control Act. The new methodology presented in this study can be used as a reference for efficient chemical management and it is expected to enable the management of a larger number of different chemicals.

## Figures and Tables

**Table 1:** Raw data of normal samples.

	Variables	X_1_	X_2_	⋯	X_j_	⋯	X_k_
Chemicals							
1		X_11_	X_21_	⋯	X_i1_	⋯	X_k1_
2		X_12_	X_22_	⋯	X_i2_	⋯	X_k2_
⋮		⋮	⋮	⋮	⋮	⋮	⋮
J		X_1j_	X_2j_	⋯	X_ij_	⋯	X_kj_
⋮		⋮	⋮	⋮	⋮	⋮	⋮
N		X_1n_	X_2n_	⋯	X_in_	⋯	X_kn_
Mean		$\bar{x_{1}}$	$\bar{x_{2}}$	⋯	$\bar{x_{i}}$	⋯	$\bar{x_{k}}$
Standard deviation		$s_{1}$	$s_{2}$	⋯	$s_{i}$	⋯	$s_{k}$

**Table 2:** Types of physical and health hazard variables according to the Globally Harmonized System (GHS).

Properties	No.	Variables	Categories									
Physical hazards	1	Explosives	1	1.1	1.2	1.3	1.4	1.5	1.6	Not Classified	Classification Not Possible	Not Applicable
	2	Flammable	1	2	3	4						
	3	Oxidizing	1	2	3							
	4	Pyrophoric	1									
	5	Self-reactive	A	B	C	D	E	F	G			
	6	Self-heating	1	2								
	7	Substances and mixtures which, when in contact with water, emit flammable gases	1	2	3							
Health hazards	8	Acute toxicity (Oral)	1	2	3	4	5					
	9	Acute toxicity (Dermal)	1	2	3	4	5					
	10	Acute toxicity (Inhalation)	1	2	3	4	5					
	11	Skin corrosion/irritation	1	2								
	12	Serious eye damage/eye irritation	1	2								
	13	Carcinogenicity	1A	1B	2							
	14	Germ cell mutagenicity	1A	1B	2							
	15	Reproductive toxicity	1A	1B	2							
	16	Specific target organ toxicity (Single exposure)	1	2	3							
	17	Specific target organ toxicity (Repeated exposure)	1	2								

**Table 3:** Process of selecting chemicals that are relatively less hazardous.

Criteria	Value
Numbers of “Category 1” classifications	0
Numbers of “Category 2” classifications	≤1
Numbers of “Category 2” for carcinogenicity, germ cell mutagenicity, and reproductive toxicity	0
Numbers of “Classification Not Possible”	≤7

**Table 4:** Results of quantifying health hazard variables.

Categories	Category 1	Category 2	Category 3	Category 4	Category 5–7	Not Classified	Classification Not Possible	Not Applicable
Score	50	45	40	35	30	25	42	1

**Table 5:** Quantification results of hazard variables using three scenarios.

Scenarios	Methods	Category 1	Category 2	Category 3	Category 4	Category 5–7	Not Classified	Classification Not Possible	Not Applicable
Scenario A ^1^	A1	50	48	46	44	42	40	47	1
	**A2**	**50**	**45**	**40**	**35**	**30**	**25**	**42**	**1**
	A3	50	42	34	26	18	10	38	1
Scenario B ^2^	B1	50	45	40	35	30	25	47	1
	B2	50	45	40	35	30	25	37	1
	B3	50	45	40	35	30	25	32	1
	B4	50	45	40	35	30	25	27	1
Scenario C ^3^	C1	50	45	40	35	30	25	42	3
	C2	50	45	40	35	30	25	42	5
	C3	50	45	40	35	30	25	42	7
	C4	50	45	40	35	30	25	42	9

^1^ Scenario A: Adjusting spacing between scores; A1: Narrowing the score gap; A2: Reference; A3: Widening the score gap ^2^ Scenario B: Adjusting scores for “Classification Not Possible”; B1: Category 1 > score > Category 2; B2: Category 3 > score > Category 4; B3: Category 4 > score > Category 5–7; B4: Category 5–7 > score. ^3^ Scenario C: Adjusting scores of “Not Applicable”; C1: score = 3; C2: score = 5; C3: score = 7; C4: score = 9.

**Table 6:** The results of Spearman correlation analysis between Mahalanobis distances (MDs) of 3028 chemicals using different quantification methods.

Scenario	Methods	A1	A2	A3	B1	B2	B3	B4	C1	C2	C3	C4
Scenario A ^1^	A1	1										
	A2	0.996	1									
	A3	0.978	0.991	1								
Scenario B ^2^	B1	0.929	0.932	0.953	1							
	B2	0.910	0.917	0.883	0.741	1						
	B3	0.660	0.669	0.628	0.465	0.876	1					
	B4	0.469	0.477	0.439	0.296	0.698	0.936	1				
Scenario C ^3^	C1	0.993	0.999	0.994	0.935	0.914	0.666	0.473	1			
	C2	0.990	0.999	0.996	0.937	0.911	0.662	0.469	0.999	1		
	C3	0.986	0.997	0.998	0.939	0.906	0.657	0.465	0.999	0.999	1	
	C4	0.981	0.994	0.999	0.940	0.901	0.652	0.460	0.996	0.998	0.999	1

^1^ Scenario A: Adjusting the spacing of scores; A1: Narrowing the score gap; A2: Reference; A3: Widening the score gap ^2^ Scenario B: Adjusting scores for “Classification Not Possible”; B1: Category 1 > score > Category 2; B2: Category 3 > score > Category 4; B3: Category 4 > score > Category 5–7; B4: Category 5–7 > score. ^3^ Scenario C: Adjusting scores for “Not Applicable”; C1: score = 3; C2: score = 5; C3: score = 7; C4: score = 9.

**Table 7:** Kappa statistics for classification methods and weighted methods.

Weighting	Estimate of Damage Radius Using ALOHA vs. MD			
	Equal Interval	Quintile	Jenks Natural Breaks	Geometrical Interval
Unweighted	0.134	0.208	0.097	0.316
Linear weighted	0.282	0.396	0.282	0.556
Quadratic weighted	0.380	0.517	0.424	0.741
